# Vaping Induced Severe Erosive Esophagitis

**DOI:** 10.1097/PG9.0000000000000327

**Published:** 2023-06-09

**Authors:** Lisa M. Pace, Katherine McGoogan

**Affiliations:** From the * Department of Pediatrics, University of Florida Health Jacksonville, Jacksonville, FL, USA; † Division of Pediatric Gastroenterology and Nutrition, Nemours Children’s Health Jacksonville, Jacksonville, FL, USA.

**Keywords:** vaping, e-cigarettes, erosive esophagitis, adolescents, gastroenterology

## Abstract

Esophagitis can be attributed to several etiologies including gastroesophageal reflux disease, caustic ingestion, medication or pill induced, radiotherapy, infectious or eosinophilic disease. There are also new consumer items on the market which can cause harmful side effects, including erosive esophagitis. In this case, we present an otherwise healthy teenage male with a history of daily vape usage with a clinical presentation of odynophagia, who was subsequently diagnosed with vaping associated esophagitis. There is currently little to no data available on the occurrence of vaping-associated esophagitis, particularly in adolescents.

## INTRODUCTION

Esophagitis refers to inflammation or injury to the esophageal mucosa. There are a vast number of causes that can lead to the development of esophagitis, the most common etiology being gastroesophageal reflux disease (GERD), which can present as erosive esophagitis (1,2). Additional etiologies include radiation therapy, infection (viral, bacterial, fungal, or parasitic), medication induced, caustic ingestion, pill esophagitis, and eosinophilic esophagitis (1,2). Despite the different potential causes for esophagitis, the clinical presentation remains similar and includes chest pain, odynophagia, or dysphagia, and possible food impaction (1). Those with severe esophagitis may develop fistulas, strictures, or perforation (1).

The epidemiology of this disease process varies based on the etiology. It is estimated that approximately 1% of the general population has erosive esophagitis (3–5). An estimated 3.6 per 100 000 experience medication-induced esophagitis and 0.35 per 100 000 experience eosinophilic esophagitis per year (1,3–5). The prevalence of infectious esophagitis is not clearly defined but is most prevalent in those who are immunocompromised, for example, patients who have human immunodeficiency virus or oncological disease (1,3–5).

The diagnosis of esophagitis can be suspected based on history and clinical examination, however, to differentiate subtypes of esophagitis an upper endoscopy and biopsy is typically performed. The endoscopic appearance of the mucosal lesions can help with diagnosis (1).

Electronic cigarettes (E-cigarettes) initially gained popularity in 2007 (6). These devices contain a vaporized nicotine solution which can be mixed with liquid flavors rather than the typical burning tobacco (6). Although initially marketed as a smoking cessation tool they have increased in popularity among adolescents (7,8). JUUL (JUUL Labs, Washington D.C) is the most common e-cigarette brand used in the U.S. JUUL pods contain 5%, or 59 mg/mL, of nicotine. This is approximately equal to the nicotine contained in 20 combustible cigarettes (8,9). In addition to the effects of nicotine, the flavoring chemicals and additional additives propose greater health risks (7–9). Testing has demonstrated multiple carcinogens in e-cigarettes including aldehydes, metals, and polycyclic aromatic hydrocarbons (8). For example, cinnamaldehyde, commonly used for cinnamon flavors, is found to be associated with impaired mucociliary function in the bronchial epithelial cells (6,8). There are currently no Food and Drug Administration (FDA) regulations in place to ensure accurate labeling of e-cigarettes (8,9).

Little to no data is currently available on the occurrence of vaping-associated esophagitis, particularly in adolescents. In this case, we present an otherwise healthy teenage male with daily vape usage with a clinical presentation of esophagitis.

## CASE PRESENTATION

The patient is a 17-year-old male with no significant medical history who presented to the emergency department after experiencing a sore throat. He noted that his initial symptoms were limited to throat pain but then progressed to odynophagia with associated dysphagia. He described his symptoms as a burning sensation with severe pain, worsened by swallowing. The patient had presented to an outside urgent care facility on day 2 of symptoms after experiencing chest pain and a foreign body sensation in the esophagus. At that time, he received rapid streptococcal antigen testing which was found to be negative and was discharged home with plans for supportive care. He reported taking a short trial of amoxicillin for 1 to 2 days and guaifenesin with no relief of symptoms. On symptom day 3 patient developed a fever with a temporal reading of 38.8 °C.

He presented to the emergency department the following day for continued progression of dysphagia and inability to tolerate solid food by mouth. He was found to be febrile (38.5 °C), tachycardiac (111 beats per minute), tachypneic (24 breaths per min), and hypertensive (132/91 mm Hg). Initial physical examination was notable for moderate soft palate erythema without lesions, bilateral tonsillar and pharyngeal erythema and swelling without exudates, and bilateral anterior cervical lymphadenopathy with mild tenderness to palpation. A 2-view chest radiograph posterior/anterior and lateral was obtained and showed mildly hyperinflated lungs with subtle peribronchial cuffing. There was no evidence of focal consolidation or foreign body. Initial labs demonstrated a complete blood count with a white blood cell count of 17.97 K/mcL and a neutrophil predominance at 90% with an elevated absolute neutrophil count of 14 800. There was no indication of anemia or thrombocytopenia. A complete metabolic panel was within normal limits. Additionally, an infectious mononucleosis screen and limited respiratory viral panel including respiratory syncytial virus, coronavirus-19, and influenza A/B were obtained and found to be negative. The patient received intravenous (IV) ketorolac, dexamethasone, and benzocaine mouthwash with minimal relief of symptoms.

Additional social history was positive for a history of vaping. The patient endorsed daily vaping approximately 3–4 times per day but stated that he had stopped vaping at the onset of his symptoms due to pain. He was sexually active with one female partner at the time of presentation. Inpatient medications included sucralfate, benzocaine mouthwash, acetaminophen-hydrocodone, ketorolac, and famotidine. On day 2–3 of admission, he continued to experience dysphagia with an inability to tolerate oral secretions secondary to pain. Physical examination changes included newly palpable posterior cervical lymphadenopathy, improved tonsillar enlargement, and ulcerations of the interior lower lip mucosa. Gastroenterology was consulted for persistence of symptoms with plans for endoscopy. During further consultation patient mentioned that he has a history of oral herpes and canker sores. Due to concerns for possible herpes simplex virus (HSV) esophagitis, he was started on prophylactic antiviral therapy with acyclovir. Human immunodeficiency virus testing was negative.

Esophagogastroduodenoscopy was performed on hospital day 4. Results showed Pediatric Grade 3 (circumferential erosive or exudative lesions) esophagitis with bleeding found throughout the entire esophagus. The most severe esophagitis was noted in the middle third of the esophagus with moderate to severe inflammation and ulceration of the proximal and distal esophagus. Histopathology showed no visualized inclusion bodies to suggest viruses and immunohistochemical stains for cytomegalovirus, HSV-1, and HSV-2 were negative. The stomach histology was positive for *Helicobacter pylori* with associated mild chronic antral and oxyntic gastritis. For this reason, he was started on quadruple therapy with amoxicillin, metronidazole, lansoprazole 20 mg twice daily, and bismuth subsalicylate.

On hospital day 6, patient had improving oral intake and was able to tolerate soft foods and liquids. He was counseled on vaping cessation. He was discharged home with plans to follow-up with gastroenterology as outpatient. Patient did not return for in-person outpatient follow-up but did report resolution of symptoms via telephone communication.

## DISCUSSION

Electronic cigarettes, originally marketed as a smoking cessation tool, have taken a huge turn in their marketing audience, and are now focused on targeting adolescents and young adults (7,9). A 2020 study conducted by Morean et al found that most teenagers were unaware that e-cigarettes (specifically JUUL) contained nicotine, while 40% did not know that a JUUL was an e-cigarette (7,9).

Currently, there are no existing FDA regulations to aid in the listing and regulation of chemicals and additives contained in vaping solutions (8). The health risks attributed to vaping include but are not limited to; impaired brain development due to exposure to high levels of nicotine, nicotine addiction, a possible gateway to marijuana use, asthma exacerbations, pneumonia, tracheitis, increased risk of cancer, E-cigarette and vaping-associated lung injury (EVALI) and although much less common, vaping can also cause erosive esophagitis (8,10,11). Additionally, prior research has shown a connection between cigarette smoking and Barrett’s esophagus secondary to GERD (12). Exposure to nicotine has also shown to cause changes in pH, thus exacerbating these symptoms (12). Given the extremely high levels of nicotine found in e-cigarettes it is plausible to conclude they may have the same effect (1,9,12).

In this case, we discussed a 17-year-old male presenting with classic symptoms of esophagitis including odynophagia, dysphagia, and chest pain. An esophagogastroduodenoscopy with biopsy was performed which demonstrated severe erosive esophagitis without evidence of infection. Given the patient’s history of daily e-cigarette usage, and negative studies for infectious etiologies, the diagnosis of vaping-associated esophagitis was concluded.

Standard treatment of erosive esophagitis typically includes removal of the offending agent or treatment of the underlying etiology and the addition of a proton pump inhibitor (6,9). In this case, pain control was provided in addition to continued IV hydration and quadruple therapy for *H. pylori* infection including a proton pump inhibitor until oral intake improved. The patient was instructed to refrain from the use of e-cigarettes for the foreseeable future.

## CONCLUSIONS

In this case, we report a rare and poorly studied gastrointestinal complication of e-cigarette and vaping use. Further evaluation and research into the possible long-term side effects and complications that can arise from the use of electronic cigarettes is still warranted. Vaping-associated esophagitis should be included in the differential diagnosis of patients with esophagitis in whom other etiologies have been excluded. Furthermore, it is important to provide counseling to our patients not only on avoiding the use of traditional cigarettes, but also the use of e-cigarettes, as their long-term consequences are still unknown.

**FIGURE 1.**
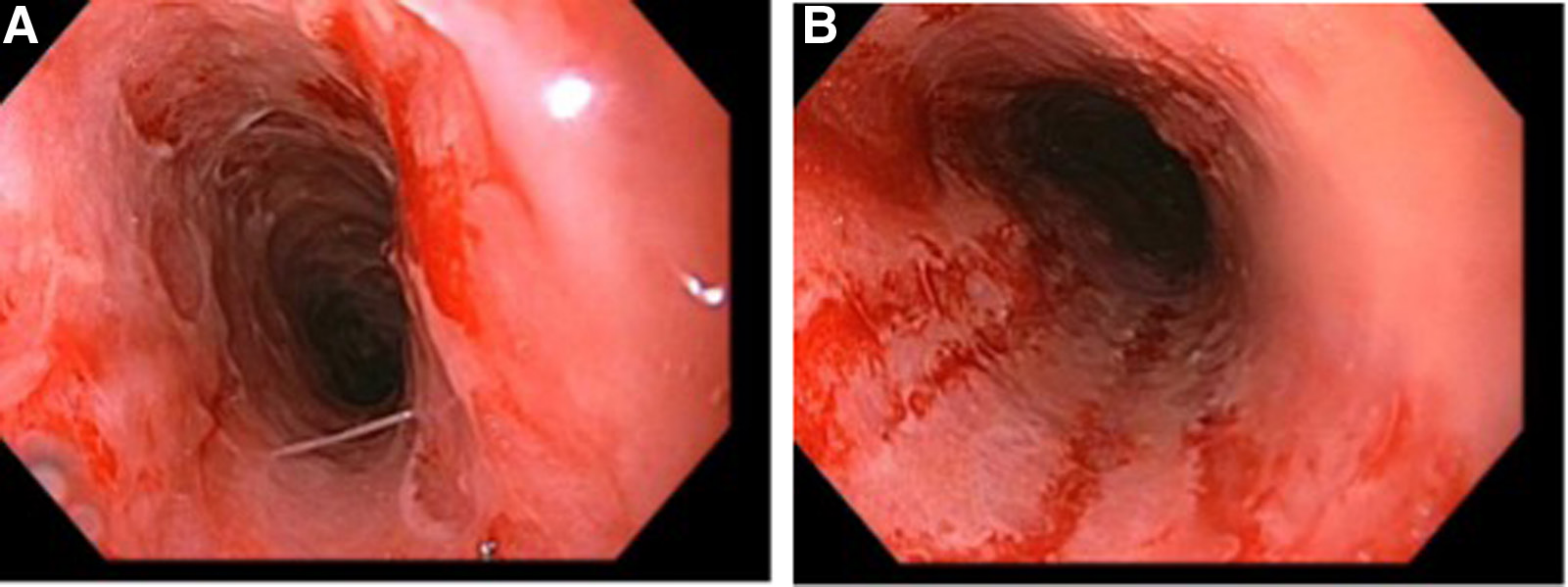
Endoscopic imagery obtained from our patient during the hospitalization confirming esophagitis. A) Endoscopic imagery demonstrating severe inflammation with ulceration of the middle esophagus. B) Endoscopic imagery demonstrating severe inflammation with circumferential erosions and bleeding of the middle esophagus.

**FIGURE 2.**
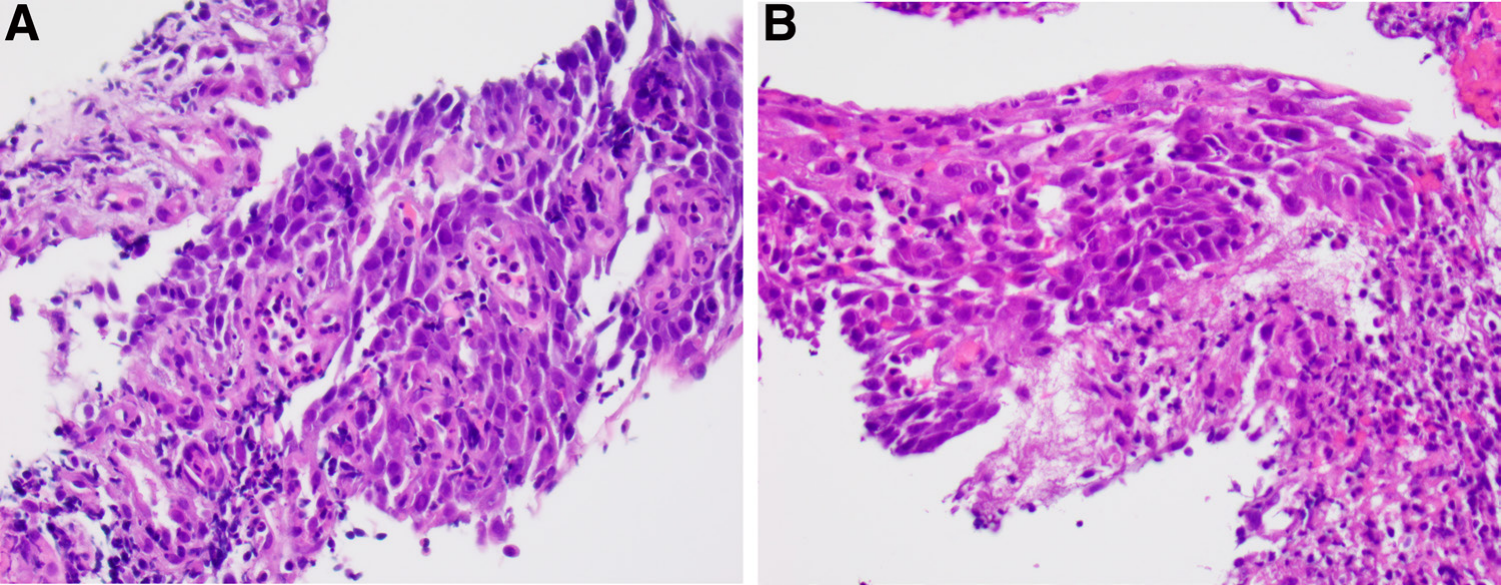
Pathological interpretation of esophageal biopsy lesions in the proximal and distal esophagus confirming diagnosis of esophagitis. A) Pathology of proximal esophagus at 40× magnification with confluent acute inflammation and areas of tissue necrosis. B, Pathology of distal esophagus at 40× magnification with confluent acute inflammation and areas of tissue necrosis. Intact squamous mucosa with infiltration by numerous acute and inflammatory cells and associated reactive changes.

## ACKNOWLEDGMENTS

Consent was obtained from the parents of the patient above to use the information provided here for a journal article, or for the purpose of a thesis or presentation.
